# Cellulose Nanofiber-Reinforced Chitosan Hydrogel Composites for Intervertebral Disc Tissue Repair

**DOI:** 10.3390/biomimetics4010019

**Published:** 2019-02-20

**Authors:** Ingo Doench, Tuan Ahn Tran, Laurent David, Alexandra Montembault, Eric Viguier, Christian Gorzelanny, Guillaume Sudre, Thibaut Cachon, Malika Louback-Mohamed, Niels Horbelt, Carlos Peniche-Covas, Anayancy Osorio-Madrazo

**Affiliations:** 1 Institute of Microsystems Engineering IMTEK, Laboratory for Sensors, University of Freiburg, 79110 Freiburg, Germany; ingo.doench@imtek.uni-freiburg.de (I.D.); tuananhtran0496@gmail.com (T.A.T.); malika.louback-mohamed@etu.univ-lyon1.fr (M.L.-M.); 2 Freiburg Materials Research Center FMF, University of Freiburg, 79104 Freiburg, Germany; 3 Ingénierie des Matériaux Polymères (IMP), CNRS UMR 5223, Université Claude Bernard Lyon 1, Université de Lyon, 69622 Villeurbanne CEDEX, France; laurent.david@univ-lyon1.fr (L.D.); alexandra.montembault@univ-lyon1.fr (A.M.); guillaume.sudre@univ-lyon1.fr (G.S.); 4 Interaction Cells Environment (ICE), VetAgro Sup, Université de Lyon, 69280 Marcy l’Etoile, France; eric.viguier@vetagro-sup.fr (E.V.); thibaut.cachon@vetagro-sup.fr (T.C.); 5 Department of Dermatology and Venerology, University Medical Center Hamburg-Eppendorf, 20246 Hamburg, Germany; c.gorzelanny@uke.de; 6 Max-Planck Institute of Colloids and Interfaces, Biomaterials Department, Science Park Golm, 14476 Potsdam, Germany; niels.horbelt@mpikg.mpg.de; 7 Center of Biomaterials, University of Havana, 10600 Havana, Cuba; cpeniche2015@yahoo.com or peniche@fq.uh.cu

**Keywords:** hydrogel biomaterial, chitosan, cellulose nanofibers, intervertebral disc, tissue engineering

## Abstract

The development of non-cellularized composites of chitosan (CHI) hydrogels, filled with cellulose nanofibers (CNFs) of the type nanofibrillated cellulose, was proposed for the repair and regeneration of the intervertebral disc (IVD) annulus fibrosus (AF) tissue. With the achievement of CNF-filled CHI hydrogels, biomaterial-based implants were designed to restore damaged/degenerated discs. The structural, mechanical and biological properties of the developed hydrogel composites were investigated. The neutralization of weakly acidic aqueous CNF/CHI viscous suspensions in NaOH yielded composites of physical hydrogels in which the cellulose nanofibers reinforced the CHI matrix, as investigated by means of microtensile testing under controlled humidity. We assessed the suitability of the achieved biomaterials for intervertebral disc tissue engineering in ex vivo experiments using spine pig models. Cellulose nanofiber-filled chitosan hydrogels can be used as implants in AF tissue defects to restore IVD biomechanics and constitute contention patches against disc nucleus protrusion while serving as support for IVD regeneration.

## 1. Introduction

Fiber-reinforced hydrogels can be engineered to mimic many biological tissues as they present similar microstructural and mechanical properties, and functionality. Thus, bioinspired composite hydrogels have great potential for tissue engineering applications. The intervertebral disc (IVD), which is the largest avascular tissue of the human body [1,2] can constitute a source of bioinspiration for the engineering of hydrogel biomaterials. The IVD connects the vertebral bodies (VBs) while keeping the spine movable. It is mainly composed of fiber-filled hydrogels and three tissue regions can be distinguished: the nucleus pulposus (NP), the annulus fibrosus (AF), and the vertebral endplates (VEPs). Proteoglycan (PG) hydrogels reinforced with collagen fibers comprise 95 wt% of the IVD [3]. The NP is a hydrated gelatinous core enclosed by the AF [3]. The water content is related to the PG content and mechanical history of the disc. It varies in the different disc regions, with the highest hydration in the NP (≈80–90 wt%) and a lower hydration in the AF (≈65 wt%). Thus, the IVD is able to withstand high compression loads and plays a key role in spine biomechanics [4,5,6]. The NP exerts a turgor, which acts as a hydrostatic pressure to support the applied load [5,6]. The AF acts as a thick-walled pressure vessel to contain the internal pressure of the nucleus [5]. With disc degeneration, the collagen and PG composition is altered, and the water content of the nucleus falls by 10–15% [7]. With loadings of above 200 N applied to the disc, for example, 20% of the disc water is driven out and the nuclear pressure falls by 36%. Intervertebral disc afflictions, which are commonly due to disc degeneration, are thought to be the leading cause of low back pain (LBP) [8]. So far, satisfactory treatments of the IVD have not been established [9], with the “gold standard” being the disc excision and fusion of the adjacent vertebral bodies (VBs). It commonly leads to further degeneration due to altered segmental motion [10]. Besides, due to the avascular character of the IVD, its self-regeneration is complicated. The development of bioinspired fiber-filled hydrogels could be the solution to support IVD regeneration and repair. Roughley et al. [11] studied the suitability of chitosan/glycerophosphate/hydroxyethyl cellulose hydrogels for the encapsulation of IVD cells and accumulation of functional extracellular matrix (ECM), mimicking that of the nucleus. Nuclear supplementation has been commonly aimed against mechanical failure unless a bioactive material also could be found to promote cell growth, PG production and disc nutrition. The treatment of the nucleus with a photocrosslinked hyaluronic acid (HA) hydrogel was proposed, which aimed to fill the NP and yield, in situ, a resistant hydrogel [12]. The NP augmentation by a gel-like formulation could prevent disc height loss and biomechanical and biochemical changes associated with degeneration.

In this work we aimed to develop cellulose nanofiber (CNF)-filled chitosan (CHI) composite hydrogels, useful for the re-establishment of the IVD biomechanics as well as for repairing and regeneration. Chitosan is a biocompatible cell growth-promoting compound and the CNFs provide mechanical reinforcement approaching the functionality of the collagen fibrils in the disc. Chitosan is the main derivative of chitin, which is mainly extracted from crustacean shells and endoskeleton of cephalopods. A lot of in vitro and in vivo studies highlight the biological properties of CHI including biodegradation [13], nontoxicity [14], cytocompatibility [15] and hemostatic activity [14]. The reported biodegradation of CHI by lysozymes could be advantageous for the design of biomaterials which should be replaced after tissue regeneration. The exceptional biocompatibility of CHI, combined with its bioactivity, explains its potential for tissue engineering [13,15,16,17,18,19]. Montembault et al. [19] prepared CHI physical hydrogels for cartilage tissue engineering, whose interaction with chondrocyte cells promoted cartilage-like ECM production in vitro. Ladet et al. [20] prepared onion-like CHI multimembrane hydrogels, suitable for engineering of multilayer tissues [18,20]. Cellulose nanofibers are renewable and constitute an environmentally friendly nanoreinforcement [21,22,23,24,25]. Cellulose nanofibers are highly available in plant biomass [25] and present outstanding mechanical performance because of their high crystallinity and aspect ratio (fiber length–width ratio) [21,26,27]. There are two main families of native CNFs [25,28]: cellulose nanowhiskers (CNWs), with the highest strength and stiffness (Young’s modulus of 114–140 GPa) [29]; and nano-/microfibrillated cellulose (MFC), which originates from the peeling of the native cellulose fibers into a network of hairy fibrils [28]. Filling of CHI matrix by polysaccharide nanofibers has been mainly reported in the development of dry film or scaffold nanocomposites [30,31,32,33,34,35,36,37]. In many of those works, instead of using unmodified cellulose fibers as proposed in the present work, the nanofibers have been modified by (2,2,6,6-tetramethylpiperidine-1-yl)oxyl (TEMPO) radical oxidation to improve the fiber–CHI matrix interactions [30,31]. Casting films of blends of CHI and poly(vinyl alcohol) (PVA) were reinforced with TEMPO- oxidized CNFs (TOCNFs) [30]. The positive effect of ultrasound irradiation on the dispersion of CNFs in CHI to produce cast films has been reported [31,36]. The question of CNF toxicity has been addressed and the low toxicity risk potential for CNFs has been reported according to ecotoxicological, cytotoxicity, and pro-inflammatory response studies [38,39]. In vitro tests carried out inhuman and murine cells have demonstrated the absence of cytotoxicity and genotoxicity for MFC suspensions [40]. In previous works, we demonstrated the good biocompatibility of CNFs in cytotoxicity evaluations in human dermal fibroblasts (HFIB-D) and human bone marrow stromal cells (HBMSCs) [41]. Cellulose nanofibers have been used as biomaterial reinforcement in tissue engineering [41,42,43]. The biocompatibility of CNFs and their non-biodegradability in the human body motivated the proposal of CNFs for the development of implants which require long-term stability to engineer tissues of high-mechanical performance (e.g., bone). Cellulose nanofiber scaffolds have been proposed for the long-term proliferation of bone-related cells [44]. In hydrogel biomaterials, the use of CNFs is promising. In addition to their mechanical performance, CNFs present high-water retention and can yield transparent biomaterials [42,45]. Moreover, CNFs can be oriented within hydrogels, by the uniaxial stretching of their composites under controlled environmental humidity. Osorio-Madrazo et al. [21] performed pioneering works where CNFs, specifically nanowhiskers, were oriented in bulk hydrogel matrices to produce reinforced anisotropic hydrogels. Thermosensitive injectable hydrogels of CHI containing TOCNFs at concentrations of 0.2%, 0.4%, 0.6%, and 0.8% (*w*/*v*) have been designed for biomedical applications [46]. These hydrogels undergo sol–gel transition at body temperature through reversible interactions between CHI and *β*-glycerophosphate. Both MC3T3-E1 pre-osteoblast cells and L929 fibroblast cells have shown biocompatibility towards CHI/TOCNF 0.4% (*w*/*v*) [46]. On one hand, the excellent biological properties of CHI, its ability to form hydrogels, the CNFs outstanding mechanical properties and demonstrated biocompatibility; and on the other hand, the fiber-reinforced hydrogel structure of the IVD motivated us to propose the development of CNF-filled CHI hydrogels for IVD tissue repair and regeneration. Different parameters, such as CHI concentration and CNF content, were considered in the processing of the composite hydrogels. The effect of these parameters in the structure and mechanical properties of the achieved hydrogel biomaterials was investigated. To assess the suitability of the obtained CNF/CHI composite hydrogels for IVD tissue repair, we performed studies in which these biomaterials were implanted in the AF tissue region of the disc to re-establish the biomechanics of lesioned discs, while serving as support for IVD regeneration and contention patch against NP protrusion. 

## 2. Materials and Method

### 2.1. Chitosan

Chitosan (Type: Chitosan144, Batch No. 20120926) from squid pen chitin was supplied by Mahtani Chitosan (Veraval, Gujarat, India). The CHI degree of acetylation (DA) was 2.5%. It was determined by ^1^H nuclear magnetic resonance (NMR) spectroscopy following the methodology of Hirai et al. [45,46]. The measurement was performed on a Bruker ALS 300 spectrometer (Bruker GmbH, Ettlingen, Germany) (300 MHz for ^1^H) at 298 K. The number- (Mn) and weight-average molar masses (Mw) of CHI were 4.10 × 10^5^ g/mol (±6.4%) and 6.11 × 10^5^ g/mol (±9.6%), respectively, with a polydispersity index of 1.49 (±10%). These molar masses were determined by size exclusion chromatography (SEC) coupled to multiangle laser light scattering (MALLS), as described elsewhere [46].

### 2.2. Cellulose Nanofibers

Gel-like suspensions of CNF were obtained from bleached pine sulfite dissolving pulp by a mechanoenzymatic method adapted from Doench et al. [41] and Pääkkö et al. [47] at the Centre Technique du Papier (CTP, Grenoble, France). Pine sulfite pulp was incubated for 1 hat 50 °C with a solution of endoglucanase FiberCare R^®^ (Novozymes Biologicals, Paris, France) at pH 5.0. The pulp was refined at 4.5% consistency with a 30.5 cm single disk refiner for 25 min. The digested samples were further refined to obtain a pulp suspension of Schopper-Riegler (SR) number higher than 80 and mean fiber length smaller than 300 µm. A quantity of 2% (*w*/*w*) fiber suspensions was processed with an Ariete homogenizer (GEA, Düsseldorf, Germany), involving one pass at 1000 bar followed by three passes at 1500 bars. The obtained CNFs exhibited a surface charge density of 40–80 mmol/kg. Thus, they were weakly charged with carboxylate moieties. The morphology of the CNFs was characterized by atomic force microscopy (AFM)with a NanoScope V controller using the NanoScope 9.1 software (Bruker Corporation, Santa Barbara, CA, USA). A droplet of a 0.001% CNF suspension was placed on a freshly cleaved mica surface. After sample drying, observations were performed in tapping mode with the AFM equipped with a tube scanner from Veeco Digital Instruments (Santa Barbara, CA, USA), using silicon tips (PPP-NCH, Nanosensors, Sindelfingen, Germany) with resonance frequency and spring constant of 360 kHz and 50 N m^−1^, respectively. 

### 2.3. Preparation of the CNF-Filled Chitosan Composite Hydrogels

A fine powder of CHI was mixed at 2 or 3% (*w*/*w*) with CNFs in water at a given CNF content (0.2%; 0.4% (*w*/*w*)). The dispersions were sonicated with a SONOPULS Ultrasonic homogenizer (Bandelin electronic GmbH, Berlin, Germany) for 5 min at 40% amplitude. Then, acetic acid was added in stoichiometric ratio to the free amine groups of the glucosamine units of CHI, to solubilize the CHI (DA = 2.5%). The mixture was kept under mechanical stirring overnight. Afterwards, the CNF/CHI viscous suspensions were neutralized with 2 M NaOH for 1 h using Petri dishes as molds. After neutralization, the gelled material could be removed from the molds and washed with distilled water untilneutral and complete removal of the salts formed during the neutralization. Finally, composite hydrogels of different CNF/CHI concentrations, of around 1–2 mm thickness, were obtained depending on the amount (1 or 2 g, respectively) of CNF/CHI viscous suspension added to the 3 × 3 cm Petri dish molds to proceed to the neutralization step. Hydrogels of CNF:CHI weight ratios of 0.1:1 and 0.2:1 with a CHI concentration of 2% (*w*/*w*); and of 0.07:1 and 0.1:1 with a CHI concentration of 3% (*w*/*w*) were produced. To characterize the dispersion of the CNFs in the CHI matrix, the freeze-dried CNF/CHI scaffolds were carefully fractured and gold sputtered in a Polaron SC 7640 (VG Microtech, East Sussex, UK), and observed by scanning electron microscopy (SEM) (Amray Inc., Bedford, MA, USA) at an accelerating voltage of 15 kV. It is worth noticing that with this technique the analysis was performed in the dry state. Nevertheless, it allowed to get insight into the dispersion of the CNFs in the dry scaffold composites, which were originated from the hydrogel processing. 

### 2.4. Microtensile Testing of CNF-Filled Chitosan Composite Hydrogels

For the tensile straining experiments, ≈2 mm wide and ≈7 mm long strips were cut out from the prepared flat nanocomposite hydrogels with a razorblade. Each sample was glued immediately onto a foliar frame with a test span of about 7 mm by using cyanoacrylate glue (Loctite^®^ 454, Germany). Afterwards, the frame with the sample was mounted onto a home-made microtensile tester surrounded by a sealed sample chamber, which allowed for controlling relative humidity (RH) during the experiment. The humidity generator (Wetsys, Setaram Instrumentation, Caluire, France) mixes dry air and water-saturated air in a controlled way to supply a gaseous flow with a stable RH that can be tuned between 5 and 95% (±0.3%) at a given temperature. At a RH of 45%, the hydrogel nanocomposite was uniaxially stretched at a constant speed of 0.2 μm/s. The RH was kept constant during the whole experiment (±2% RH). The applied force (*F*) was measured by a load cell with a maximum capacity of 50 N. The displacement of the motorized linear stage was recorded. Nominal stress *σ* = *F*/(*w*_0_*t*_0_) and nominal strain *ε* = (*l* − *l*_0_)/*l*_0_ values were estimated with *w*_0_ being the initial width, *t*_0_ the initial thickness, *l* the actual length, and *l*_0_ the initial length of the cut hydrogel strip. At least three different strips of a given sample were tested under similar experimental conditions. It is worth noticing that the true stress of the sample should be different from the calculated one because of a reduction of the sample cross-sectional area (*wt*) caused by straining and/or partial drying.

### 2.5. Suitability of the CNF/CHI Composite Hydrogels for IVD Repair and the Re-Establishment of IVD Biomechanics

#### 2.5.1. Implant of CNF/CHI Composite Hydrogels in the Annulus Fibrosus Region of the IVD. Biomechanical Studies

**Figure 1:**
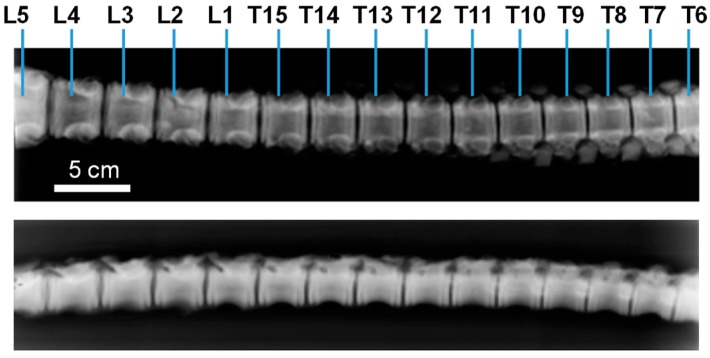
Radiographies showing the frontal (above) and lateral (below) views of the thoracic (T) and lumbar (L) parts of the spine of the growing-finishing pig model used in the study, with the different motion segments considered in the biomechanical testing.

The design of a bioactive hydrogel to seal, reinforce, and repair the AF disc region would ensure appropriate long-term tissue regeneration while providing a mechanical support to avoid post-surgery hernia reoccurrence. To this end, the determination of spine mechanical loading and motion is challenging in biomechanics. To understand its functionality, the needed smallest unit (motion segment) consists of two vertebrae and the disc joining them together [6]. Around 30 kg growing-finishing pigs were used as models for compression testing of healthy and damaged discs, these latter repaired with the composite hydrogels. Discs localized between the lumbar L5 and thoracic T7 vertebrae were considered (Figure 1). Motion segments were dissected, consisting of two adjacent VEPs and the in-between disc. A total of 9 motion segments were tested: T8–T9, T10–T11, T11–T12, T12–T13, T14–T15, L1–L2, L2–L3, L3–L4, and L4–L5 discs (Figure 1).

**Figure 2:**
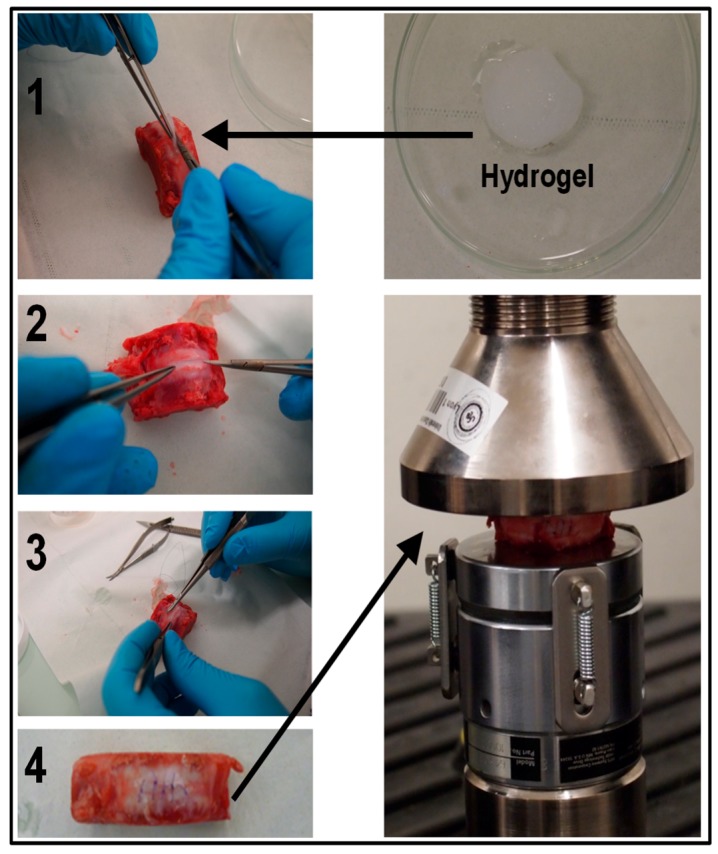
Implantation of the CNF/CHI composite hydrogel shown in the top-right (3% CHI/0.4% CNF (*w*/*w*)) in an annulus fibrosus tissue defect (**1**,**2**) of a L4–L5 lumbar disc of a pig model (Figure 1). The disc was sutured at the implanted position (**3**,**4**) and subjected to compression loading as shown in the bottom-right of the figure.

The compression tests have been performed on a Shimadzu Autograph AG-X plus (Kyoto, Japan) equipped with a 10 kN load cell. After a pre-loading of 200 N [7], compression was applied until failure. Concerning lesioned discs, a fenestration of length ≈10 mm, width ≈5 mm, and thickness ≈1.5 mm was performed in the ventral part of the AF. A composite hydrogel (3% CHI/0.4% CNF (*w*/*w*)) piece of similar dimensions as the performed fenestration was carefully implanted within the AF defect. Then, the disc was sutured at the implanted position and subjected to compression loading. Figure 2 shows the methodology followed to prepare and mechanically test a fenestrated disc that was implanted with the hydrogel.

All animal subjects used in this study abide to the French and the National Institutes of Health Guide for the Care and Use of Laboratory Animals, by completing the legal forms and having all the required authorizations (legal and ethical). The experimentations on animal subjects were performed by authorized staff in labelled facilities (Institute Claude Bourgelat, VetAgro Sup, Lyon Veterinary School, France) according to ethical agreement (Proposal Number 69.127.05.05, Ethical Committee Agreement Number 1440).

#### 2.5.2. Cell Culture of NIH/3T3 Fibroblasts on CNF/CHI Hydrogels

To evaluate the suitability of the CNF/CHI composite hydrogels for IVD tissue engineering, experiments were performed with fibroblast cells cultured on the hydrogels of different CHI concentrations and CNF contents. Cell cultures of 3T3 cells developed using the NIH Swiss mouse embryo fibroblasts were performed (murine fibroblast, strain: NIH/Swiss). NIH/3T3 fibroblast cells were grown in T75 (75 cm^2^) cell culture flasks (Sarstedt, Nümbrecht, Germany). The cells were cultured in Dulbecco’s modified Eagle medium (DMEM) supplemented with 2 mM l-glutamine and 10% fetal bovine serum (FBS) (Gibco, Thermo Fisher Scientific, Leicestershire, UK) at 37 °C in a humidified atmosphere of 5% CO_2_ for 1 week. Upon 90% confluence, cells were rinsed twice with phosphate-buffered saline (PBS) (Gibco, Thermo Fisher Scientific, Leicestershire, UK) followed by detachment with trypsin/ethylenediaminetetraacetic acid (EDTA) for 5 min and neutralization with the corresponding cell culture medium. After detachment, cells were spun down in a centrifuge for 5 min at 110 rcf (Rotor F-45-30-11, Eppendorf 5417R, Hamburg, Germany). The supernatant was discarded and cells were diluted into the culture medium. The CNF/CHI hydrogel pieces were put in 24-well cell culture plates, covering the whole well surface for subsequent use for cell growth. Firstly, the hydrogel pieces were incubated with 1 mL of culture medium for 30 min. Then, the medium was removed and cells were transferred to each well in wished dilution. A suspension of the cells in culture medium (500 μL) was added on the hydrogel surface and NIH/3T3 cells were seeded at 200,000 cells per well on average in triplicates. Cells seeded in empty wells (i.e., without the hydrogel) were used as control.

## 3. Results and Discussion

### 3.1. Structure of the CNF/CHI Hydrogels

**Figure 3:**
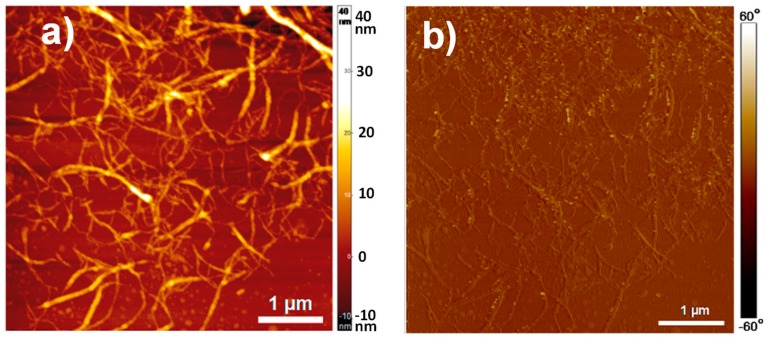
(**a**) Atomic force microscopy topography and (**b**) phase images of cellulose nanofibers used to reinforce the CNF-filled CHI composite hydrogels.

Figure 3 shows atomic force microscopy (AFM) images of the CNFs used as nanoreinforcement of the CNF/CHI composite hydrogels. They reveal an entangled network of interconnected nanofibrils with average width of 35.2 ± 8.1 nm and bundles up to 100 nm width. The relative larger width of the fibrils observed by this method could be due to the drying of the fibrils inducing the formation of aggregates [47]. The mechanoenzymatic hydrolysis during the CNFs processing leaves long nanoscale fibrils, preserving the native cellulose I crystalline allomorph with partly amorphous regions. Thus, nanofibrils are able to inherently entangle [47]. Such long entangled nanofibers may be feasible for nanoreinforcement percolation in the composite hydrogels.

**Figure 4:**
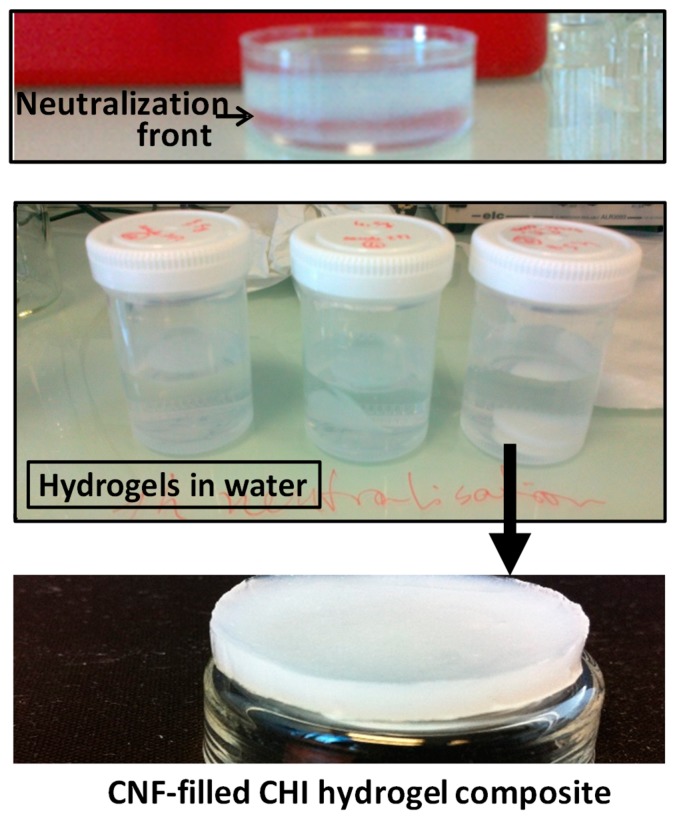
Images of the material appearance during the different steps of the processing of CNF-filled CHI composite hydrogels. (Top) Neutralization of a CNF/CHI viscous suspension with aqueous NaOH, showing the neutralization front which moves with time from the top to the bottom of the Petri dish. (Middle) Three examples of flat composite hydrogels (2% CHI/0.2% CNF (*w*/*w*)) preserved in distilled water after finalized the washing steps.

Figure 4 shows examples of CNF/CHI composite hydrogels obtained after neutralization of CNF-filled CHI viscous suspensions in Petri dish molds and further washing in distilled water.

**Figure 5:**
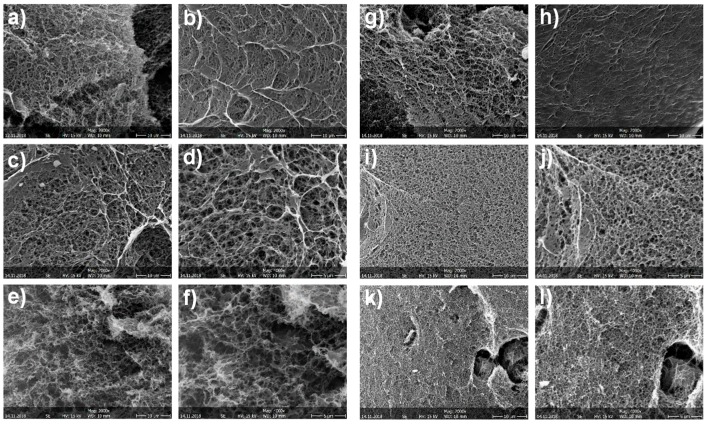
Scanning electron micrographs of scaffolds obtained after freeze-drying of CNF/CHI composite hydrogels containing (**a**–**f**) 2% and (**g**–**l**) 3% (*w*/*w*) chitosan. (**a**,**b**) neat 2% CHI; (**c**,**d**) 2% CHI/0.2% CNF; (**e**,**f**) 2% CHI/0.4% CNF; (**g**,**h**) neat 3% CHI; (**i**,**j**) 3% CHI/0.2% CNF; (**k**,**l**) 3% CHI/0.4% CNF. (**a**,**c**–**g**,**i**–**l**) Fracture surfaces; (**b**,**h**) top view SEM micrographs of the freeze-dried CHI hydrogel surface. Scale bars:10 µm (**a**–**c**,**e**,**g**–**i**,**k**); 5 µm (**d**,**f**,**j**,**l**).

Figure 5 shows SEM micrographs of CNF/CHI hydrogels of different compositions which were freeze-dried for observation. The scaffolds revealed a sponge-like network microstructure with interconnected pores for all CNF/CHI concentrations considered in the hydrogel preparation. In all considered compositions, a regular network structure with indistinguishable cellulose nanofibers was observed, which can be due to the good interfacial compatibility of the polysaccharide nanofibers and matrix allowing for good dispersion of the CNFs in the composite. Scanning electron microscopy (SEM)images reveal that with the increase in CHI concentration the porosity decreases. For a given CHI concentration, the increase in CNF content yielded biomaterials of smaller pore sizes. In a given hydrogel sample, observations at the hydrogel outer top surface (Figure 5b,h) (which was directly in contact with the NaOH solution during neutralization) differed from those of surface fractures corresponding to the inner part of the hydrogel (Figure 5a,g). At the outer top surface, a smoother microstructure was observed with a pore size smaller (Figure 5b,h) than in the inner hydrogel (Figure 5a,g). It could be explained due to the hydrogel processing with a directional diffusion of the base (NaOH) during the neutralization, in which the neutralization front moves from the top to the bottom of the Petri dish mold (Figure 4). Sereni et al. [48] also reported on porosity variations throughout the cross-section of CHI physical hydrogels due to the preparation procedure similar to that used in this work to prepare composite hydrogels.

### 3.2. Micromechanical Properties of the CNF/CHI Composite Hydrogels

**Figure 6:**
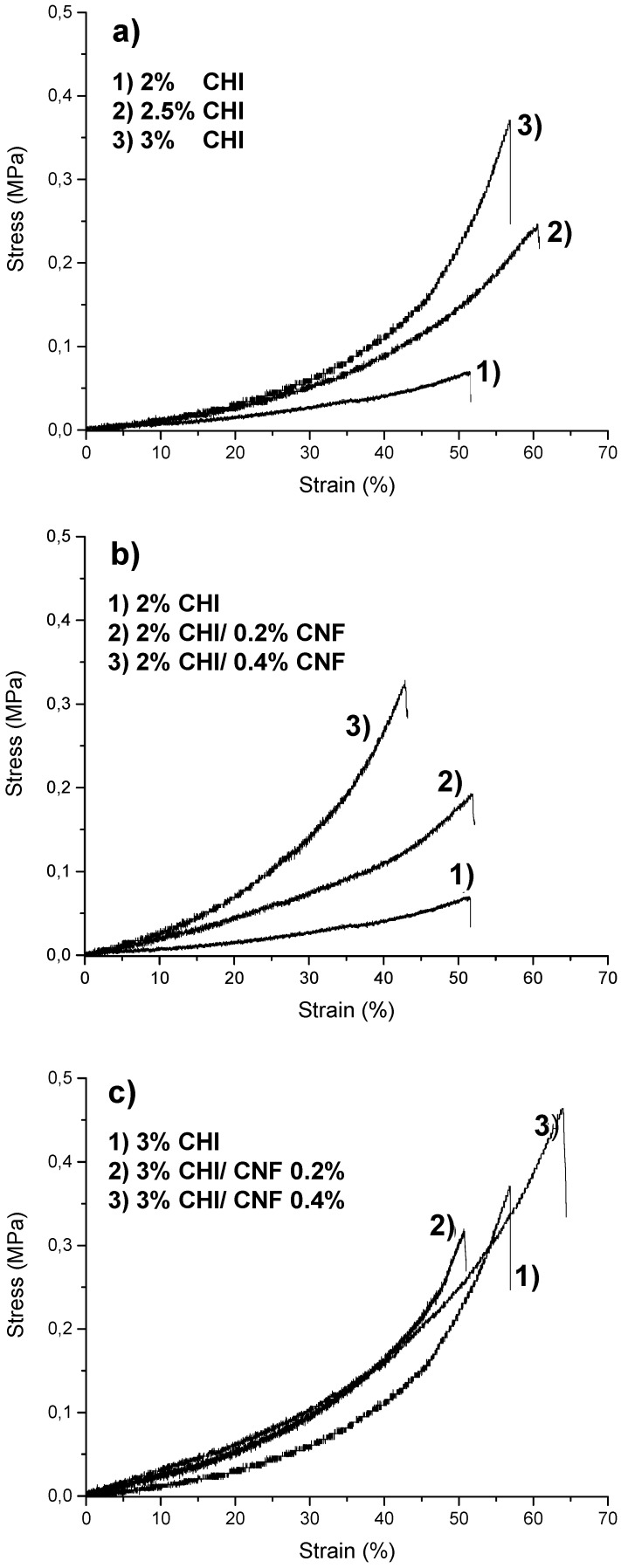
Stress–strain curves of CNF/CHI hydrogels of different compositions analyzed in microtensile tests performed under controlled 45% relative humidity.

Mechanical properties characterization of the composite hydrogels is shown in Figure 6. Micromechanical studies were performed under controlled RH. Figure 6 shows the impact of the increase of both CHI concentration and CNF content on the hydrogel mechanical properties. Table 1 gives the elastic modulus (*E*) values estimated from the slope of the tangent comprising the range between the origin and the lowest strain values of the stress–strain curves (Figure 6). Strain at break values of 45–65% and *E* between 0.073 and 0.30 MPa were obtained depending on the hydrogel composition. As expected, the increase of the CNF content enhanced the hydrogel mechanical properties. The highest value of *E* was obtained for composites containing a CHI concentration of 3% (*w*/*w*) and a CNF content of 0.4% (*w*/*w*). Results were in the range of relatively good mechanical performance for polysaccharide hydrogel biomaterials [21,49]. These results indicate that the nanofibers provided reinforcement to the hydrogel matrix of both 2 and 3% (*w*/*w*) CHI. The surface of CNFs used in this work was weakly charged with carboxylate moieties displaying a surface charge density of 40–80 mmol/kg [50,51]. During hydrogel processing, in the viscous suspensions electrostatic interactions could be established between CHI (a polycation) and CNFs (a polyanion). Hydrogen bond interactions also could be established between the –OH and –NH functionalities of CHI and the –OH groups of cellulose. Besides, hydrophobic interactions together with H-bonding contribute to the formation of the hydrogel network during the neutralization step. All mentioned interactions contribute to the good compatibility between the nanofibers and the CHI matrix and may allow for stress transfer from the CHI hydrogel matrix to the nanofibers, resulting in improved mechanical properties of the composites. Chitosan chains may absorb on the surface of CNF and play a role in the bridging of nanofibers [41,52,53]. 

### 3.3. Suitability of the CNF/CHI Composite Hydrogels for IVD Repair and Biomechanics Re-Establishment

**Figure 7:**
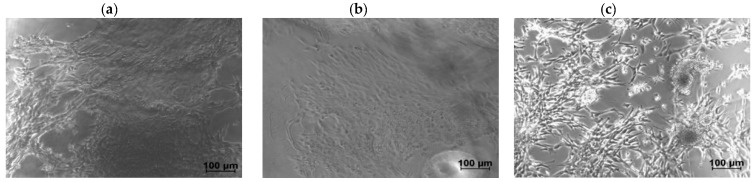
Phase-contrast images of NIH/3T3 fibroblasts after six days of culture on a 2% CHI/0.2% CNF composite hydrogel. (**a**) Cells cultured on original top hydrogel surface; (**b**) cells cultured on freshly cut hydrogel surface; (**c**) cells cultured without hydrogel (control).

Examples of optical phase-contrast images of NIH/3T3 fibroblasts after six days of culture on a CNF/CHI hydrogel composite are shown in Figure 7. After 48 h (data not shown), confluent cell spreading was practically achieved on both the neat 2% (*w*/*w*) CHI hydrogels and the corresponding CNF/CHI composites. The differences in cellular behavior observed for different CHI concentrations were mainly explained by the increase of stiffness and density (porosity decrease) of the hydrogel when increasing CHI concentration. Higher CHI concentrations led to less accessibility for the cells, reduced adhesion, and longer time was needed for representative spreading. Ata given CHI concentration, for the CNF contents considered in this study (0.2–0.4% (*w*/*w*)), similar cell behaviors were observed when using CHI alone or CNF-filled CHI hydrogels. A remarkable aspect was that for CHI concentrations higher than 2% (*w*/*w*), as considered in this study, the fibroblasts mostly grew on the hydrogel surface, which hardly revealed a three-dimensional (3D) cell culture appearance (Figure 7a). When the top surface of the hydrogel was cut to produce a fresh surface revealing the inner microstructure of the flat hydrogel (i.e., farther from the original top surface), proliferation of the cells within the hydrogel structure was observed, as revealed in Figure 7b. This relatively low proliferation in the bulk of the hydrogels for CHI concentration higher than 2% could be also related to variations in the density and stiffness throughout the hydrogel cross-section (when moving from the top to the inner part). Actually, in the hydrogel processing, a directional neutralization occurred since the neutralization front moves from the top to the bottom of the Petri dish mold (Figure 4). Sereni et al. [48] reported on porosity variations in the cross-section of CHI hydrogels due to the preparation procedure, which was similar to that used in this study. This effect may also be present in CNF-filled CHI hydrogels, which indeed could influence the fibroblast behavior on the hydrogel.

**Figure 8:**
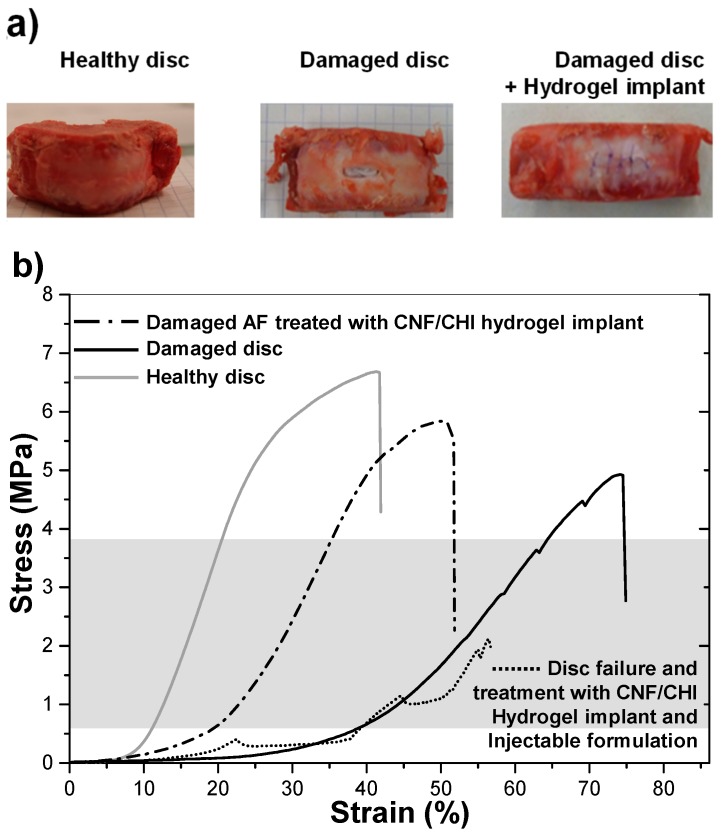
(**a**) Examples of healthy, damaged, and hydrogel-implanted discs for which spinal motion segments, consisting of two vertebral endplates and the disc joining them together, biomechanical testing were performed. (**b**) Compression stress–strain curves of healthy, damaged, and hydrogel-implanted intervertebral disc. For the latter, the intervertebral disc annulus fibrosus was repaired with a 3% CHI/0.4% CNF composite hydrogel patch. Grey shadow: Physiological loading range.

Using CNF/CHI hydrogel patches for the repair of the AF disc region is justified by the reported modeling of IVD tissue, in which the fibrous network of AF plays a critical role in the IVD biomechanics [54]. A rigorous material definition maybe achieved to incorporate the role of fiber–matrix interactions in the complex IVD tissue. The design of hydrogel composite implants needs to reach adequate mechanics and relative long-term performance as well as fix onto the tissue defect (e.g., by adhesion or suture). The design of a bioactive hydrogel to seal, reinforce, and repair the AF, as proposed here, may ensure appropriate long-term tissue regeneration, while providing a mechanical support to avoid post-surgery hernia reoccurrence or compressive scar. We studied the input of the engineered CNF/CHI composite hydrogels on the biomechanics of the IVD. The hydrogel composition with the best mechanical properties achieved in the microtensile testing results (3% CHI/0.4% CNF) was selected for implantation studies. Figure 8 shows the resulting nonlinear stress–strain relationship of the motion segments’ mechanical behavior. The curves of the healthy, damaged, and hydrogel-implanted discs showed an initial neutral zone (NZ), where the discs undergo relatively large deformation under low applied force. Then, the elastic zone (EZ) was reached. This linear EZ corresponds to the physiological loading range (Figure 8, grey shadow) with stresses reported to be 0.6–3.8 MPa, related to body weight or exerted muscle forces during bending and torsion (0.6–1.3 MPa) [5], and typical or extreme daily loadings like object lifting and vibrating vehicle driving (2.5–3.8 MPa) [7,55,56]. The EZ of the healthy disc revealed the physiological loading region and a pronounced slope. The positive input of the hydrogel implantation in AF defect on disc biomechanics is also shown. While both curves of the damaged and hydrogel-treated discs revealed an EZ within the physiological loading range, the linear EZ of the curve of the hydrogel-treated disc revealed a higher slope similar to the healthy disc. Moreover, the stress at failure of the healthy disc, hydrogel-implanted disc, and the untreated damaged disc (6.7, 5.9, and 5.0 MPa, respectively) demonstrates the beneficial role of hydrogel composite implantation to re-establish IVD biomechanics.

**Figure 9:**
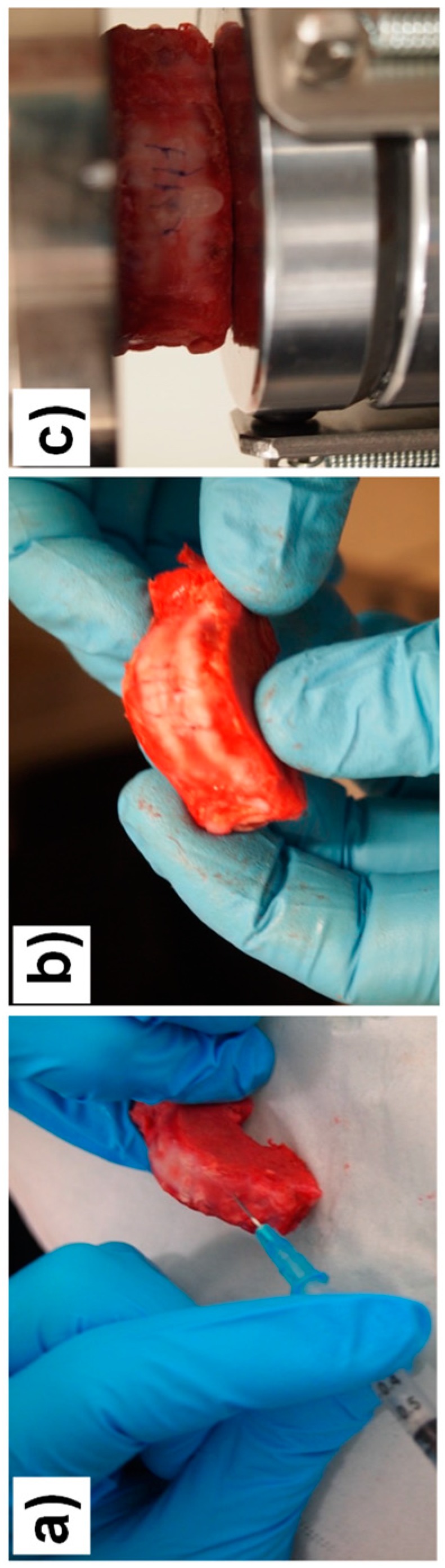
Appearance of discs after complete failure, which were subject to a treatment combination of CNF/CHI hydrogel (“gel state”) implantation in the disc annulus fibrosus region (Figure 2) and (**a**) injection of CNF/CHI viscous suspension (“sol state”) in the nucleus pulposus region. (**b**) Appearance after failure in a second mechanical testing, after already achieved failure in a previous compression test (Figure 8); the integrity of the disc was preserved at the hydrogel-implanted disc side. (**c**) The implanted hydrogel could not withstand a fluid leak during nucleus prolapse.

The range of motion (ROM)—the displacement at the largest applied load—of the hydrogel-implanted disc approached more that of the healthy disc, than did the damaged disc. In contrast to the damaged disc, the shape of the curve of the hydrogel-implanted disc was much closer to that of the healthy disc. Similarly to what is observed for the healthy disc, the hydrogel-implanted disc revealed a plastic deformation range after the EZ. The dotted curve in the Figure 8 corresponds to a hydrogel-implanted disc, which initially reached complete failure, but was retreated with a new hydrogel patch in the AF defect and injected with 120 µL of CNF/CHI viscous formulation (sol state) in the nucleus [41]. With this combination of hydrogel implant and injectable treatment, a stress of 2.2 MPa, still in the range of physiological loading, could be achieved. Pictures of discs subjected to this treatment combination after complete failure are shown in Figure 9. In a first disc, when the failure was achieved for the second time, the integrity of the disc was preserved at the implanted side (Figure 9b), revealing the ability of the hydrogel implant to withstand any fluid leak during nucleus prolapse. In a second disc, the implanted hydrogel could not withstand fluid leak during nucleus prolapse (Figure 9c). Noticeably, the images in Figure 9 correspond to the appearance after mechanical testing of motion segments which already achieved failure in a previous compression testing. 

Research on hydrogel biomaterial therapies for IVD tissue engineering is facing many challenges. Our designed materials appear compatible with early mechanical stimulation, which also appears to affect the behavior of chondrocytes and thus the self-regeneration of cartilaginous tissues [54]. A proportional relationship was found between compression of a chondrocyte-loaded tissue engineered biomaterial and the culture time-dependent increase of stiffness of the biomaterial [54,57,58].

## 4. Conclusions

Cellulose nanofiber-filled chitosan composite hydrogels were developed and structurally and mechanically characterized. The addition of CNF contributes to the improvement of the mechanical properties of CHI hydrogel matrices. From ex vivo experiments performed in pig vertebral unit models, we showed that the implantation of CNF-reinforced CHI hydrogels in AF disc defects contributes to the restoration of the disc biomechanics by approaching the functionality of a healthy disc. Furthermore, these hydrogel composites can serve as contention patches against nucleus protrusion and also as hydrogel biomaterial to support cell growth in a 3D environment, and finally show characteristics and properties which are promising for IVD tissue engineering.

## Figures and Tables

**Table 1:** Elastic modulus (*E*) values of CNF/CHI hydrogels of different compositions obtained by microtensile test.

Hydrogel Composition	*E* (MPa) ^1^
2% CHI	0.073 ± 0.0024
3% CHI	0.13 ± 0.0045
2% CHI/0.2% CNF	0.22 ± 0.0013
3% CHI/0.2% CNF	0.24 ± 0.0013
2% CHI/0.4% CNF	0.28 ± 0.0021
3% CHI/0.2% CNF	0.30 ± 0.0011

^1^ Data are shown as the mean ± standard deviation.

## References

[1] Urban J.P.G., Smith S., Fairbank J.C.T. (2004). Nutrition of the intervertebral disc. Spine.

[2] Chan S.C.W., Gantenbein-Ritter B. (2012). Intervertebral disc regeneration or repair with biomaterials and stem cell therapy—Feasible or fiction?. Swiss Med. Wkly..

[3] Whatley B.R., Wen X. (2012). Intervertebral disc (IVD): Structure, degeneration, repair and regeneration. Mater. Sci. Eng. C.

[4] Rapoff A.J., Zdeblick T.A., Yoganandan N., Pintar F.A., Larson S.J., Sances A.J. (1998). Biomechanical models of the cervical spine. Frontiers in Head and Neck Trauma: Clinical and Biomechanical.

[5] Sato K., Kikuchi S., Yonezawa T. (1999). In vivo intradiscal pressure measurement in healthy individuals and in patients with ongoing back problems. Spine.

[6] Hukins D.W.L., Meakin J.R. (2000). Relationship between structure and mechanical function of the tissues of the intervertebral joint. Am. Zool..

[7] Adams M.A., McNally D.S., Dolan P. (1996). ‘Stress’ distributions inside intervertebral discs. The effects of age and degeneration. The Journal of bone and joint surgery. J. Bone Joint Surg. Br..

[8] Sharifi S., Bulstra S.K., Grijpma D.W., Kuije R. (2015). Treatment of the degenerated intervertebral disc; closure, repair and regeneration of the annulus fibrosus. J. Tissue Eng. Regen. Med..

[9] Gore M., Sadosky A., Stacey B.R., Tai K.-S., Leslie D. (2012). The burden of chronic low back pain: Clinical comorbidities, treatment patterns, and health care costs in usual care settings. Spine.

[10] van Ooij A., Oner F.C., Verbout A.J. (2003). Complications of artificial disc replacement: A report of 27 patients with the SB Charité disc. J. Spinal Disord. Tech..

[11] Roughley P., Hoemann C., DesRosiers E., Mwale F., Antoniou J., Alini M. (2006). The potential of chitosan-based gels containing intervertebral disc cells for nucleus pulposus supplementation. Biomaterials.

[12] Chou A.I., Nicoll S.B. (2009). Characterization of photocrosslinked alginate hydrogels for nucleus pulposus cell encapsulation. J. Biomed. Mater. Res. A.

[13] Gorzelanny C., Pöppelmann B., Pappelbaum K., Moerschbacher B.M., Schneider S.W. (2010). Human macrophage activation triggered by chitotriosidase-mediated chitin and chitosan degradation. Biomaterials.

[14] Rao S.B., Sharma C.P. (1997). Use of chitosan as a biomaterial: Studies on its safety and hemostatic potential. J. Biomed. Mater. Res..

[15] Chatelet C., Damour O., Domard A. (2001). Influence of the degree of acetylation on some biological properties of chitosan films. Biomaterials.

[16] Mathews S., Gupta P.K., Bhonde R., Totey S. (2011). Chitosan enhances mineralization during osteoblast differentiation of human bone marrow-derived mesenchymal stem cells, by upregulating the associated genes. Cell Prolif..

[17] Deng Y., Ren J., Chen G., Li G., Wu X., Wang G., Gu G., Li J. (2017). Injectable in situ cross-linking chitosan-hyaluronic acid based hydrogels for abdominal tissue regeneration. Sci. Rep..

[18] Ladet S.G., Tahiri K., Montembault A.S., Domard A.J., Corvol M.T.M. (2011). Multi-membrane chitosan hydrogels as chondrocytic cell bioreactors. Biomaterials.

[19] Montembault A., Tahiri K., Korwin-Zmijowska C., Chevalier X., Corvol M.-T., Domard A. (2006). A material decoy of biological media based on chitosan physical hydrogels: Application to cartilage tissue engineering. Biochimie.

[20] Ladet S., David L., Domard A. (2008). Multi-membrane hydrogels. Nature.

[21] Osorio-Madrazo A., Eder M., Rueggeberg M., Pandey J.K., Harrington M.J., Nishiyama Y., Putaux J.-L., Rochas C., Burgert I. (2012). Reorientation of cellulose nanowhiskers in agarose hydrogels under tensile loading. Biomacromolecules.

[22] Osorio-Madrazo A., David L., Peniche-Covas C., Rochas C., Putaux J.-L., Trombotto S., Alcouffe P., Domard A. (2015). Fine microstructure of processed chitosan nanofibril networks preserving directional packing and high molecular weight. Carbohydr. Polym..

[23] Osorio-Madrazo A., David L., Trombotto S., Lucas J.-M., Peniche-Covas C., Domard A. (2011). Highly crystalline chitosan produced by multi-steps acid hydrolysis in the solid-state. Carbohydr. Polym..

[24] Osorio-Madrazo A., Laborie M.-P., Dufresne A., Thomas S., Pothen L.A. (2013). Morphological and thermal investigations of cellulosic bionanocomposites. Biopolymer Nanocomposites.

[25] Samyn P., Osorio-Madrazo A., Barhoum A., Bechelany M., Makhlouf A. (2018). Native crystalline polysaccharide nanofibers: Processing and properties. Handbook of Nanofibers.

[26] Mao J., Osorio-Madrazo A., Laborie M.-P. (2013). Preparation of cellulose I nanowhiskers with a mildly acidic aqueous ionic liquid: Reaction efficiency and whiskers attributes. Cellulose.

[27] Favier V., Chanzy H., Cavaillé J.Y. (1995). Polymer nanocomposites reinforced by cellulose whiskers. Macromolecules.

[28] Klemm D., Kramer F., Moritz S., Lindström T., Ankerfors M., Gray D., Dorris A. (2011). Nanocelluloses: A new family of nature-based materials. Angew. Chem. Int. Ed Engl..

[29] Šturcová A., Davies G.R., Eichhorn S.J. (2005). Elastic modulus and stress-transfer properties of tunicate cellulose whiskers. Biomacromolecules.

[30] Choo K., Ching Y., Chuah C., Julai S., Liou N.-S. (2016). Preparation and characterization of polyvinyl alcohol–chitosan composite films reinforced with cellulose nanofiber. Materials.

[31] Ghazanfari M., Ranginkar Jahromi I., Moallemi-Oreh A., Ebadi-Dehaghani H., Akbarzadeh M. (2016). Evaluation of mixing efficiency in elaborating of chitosan/cellulose nanocomposite via statistical analyses. Int. J. Biol. Macromol..

[32] de Mesquita J.P., Donnici C.L., Pereira F.V. (2010). Biobased nanocomposites from layer-by-layer assembly of cellulose nanowhiskers with chitosan. Biomacromolecules.

[33] El Miri N., Abdelouahdi K., Zahouily M., Fihri A., Barakat A., Solhy A., El Achaby M. (2015). Bio-nanocomposite films based on cellulose nanocrystals filled polyvinyl alcohol/chitosan polymer blend. J. Appl. Polym. Sci..

[34] Li H.-Z., Chen S.-C., Wang Y.-Z. (2015). Preparation and characterization of nanocomposites of polyvinyl alcohol/cellulose nanowhiskers/chitosan. Compos. Sci. Technol..

[35] Abdul Khalil H.P.S., Saurabh C.K., Adnan A.S., Nurul Fazita M.R., Syakir M.I., Davoudpour Y., Rafatullah M., Abdullah C.K., Haafiz M.K.M., Dungani R. (2016). A review on chitosan-cellulose blends and nanocellulose reinforced chitosan biocomposites: Properties and their applications. Carbohydr. Polym..

[36] Celebi H., Kurt A. (2015). Effects of processing on the properties of chitosan/cellulose nanocrystal films. Carbohydr. Polym..

[37] Liu M., Zheng H., Chen J., Li S., Huang J., Zhou C. (2016). Chitosan-chitin nanocrystal composite scaffolds for tissue engineering. Carbohydr. Polym..

[38] Kovacs T., Naish V., O’Connor B., Blaise C., Gagne F., Hall L., Trudeau V., Martel P. (2010). An ecotoxicological characterization of nanocrystalline cellulose (NCC). Nanotoxicology.

[39] Pértile R.A., Moreira S., Gil R.M., Correia A., Guãrdao L. (2012). Bacterial cellulose: Long-term biocompatibility studies. J. Biomater. Sci. Polym..

[40] Pitkänen M. Nanofibrillar Cellulose—In Vitro Study of Cytotoxic And Genotoxic Properties. Proceedings of the 2010 International Conferene on Nanotechnology for the Forest Products Industry.

[41] Doench I., Torres-Ramos M.E., Motembault A., de Oliveira P., Halimi C., Viguier E., Heux L., Siadous R., Thiré R., Osorio-Madrazo A. (2018). Injectable and gelable chitosan formulations filled with cellulose nanofibers for intervertebral disc tissue engineering. Polymers.

[42] Eyholzer C., Borges de Couraça A., Duc F., Bourban P.E., Tingaut P., Zimmermann T., Månson J.A.E., Oksman K. (2011). Biocomposite hydrogels with carboxymethylated, nanofibrillated cellulose powder for replacement of the nucleus pulposus. Biomacromolecules.

[43] Borges A.C., Eyholzer C., Duc F., Bourban P.-E., Tingaut P., Zimmermann T., Pioletti D.P., Månson J.-A.E. (2011). Nanofibrillated cellulose composite hydrogel for the replacement of the nucleus pulposus. Acta Biomater..

[44] Kim H.J., Oh D.X., Choy S., Nguyen H.-L., Cha H.J., Hwang D.S. (2018). 3D cellulose nanofiber scaffold with homogeneous cell population and long-term proliferation. Cellulose.

[45] De France K.J., Hoare T., Cranston E.D. (2017). Review of hydrogels and aerogels containing nanocellulose. Chem. Mater..

[46] Nguyen T.H.M., Abueva C., Ho H.V., Lee S.-Y., Lee B.-T. (2018). In vitro and in vivo acute response towards injectable thermosensitive chitosan/TEMPO-oxidized cellulose nanofiber hydrogel. Carbohydr. Polym..

[47] Pääkkö M., Ankerfors M., Kosonen H., Nykänen A., Ahola S., Österberg M., Ruokolainen J., Laine J., Larsson P.T., Ikkala O. (2007). Enzymatic hydrolysis combined with mechanical shearing and high-pressure homogenization for nanoscale cellulose fibrils and strong gels. Biomacromolecules.

[48] Sereni N., Enache A., Sudre G., Montembault A., Rochas C., Durand P., Perrard M.-H., Bozga G., Puaux J.-P., Delair T. (2017). Dynamic structuration of physical chitosan hydrogels. Langmuir.

[49] Sharifi S., van Kooten T.G., Kranenburg H.-J.C., Meij B.P., Behl M., Lendlein A., Grijpma D.W. (2013). An annulus fibrosus closure device based on a biodegradable shape-memory polymer network. Biomaterials.

[50] Foster E.J., Moon R.J., Agarwal U.P., Bortner M.J., Bras J., Camarero-Espinosa S., Chan K.J., Clift M.J.D., Cranston E.D., Eichhorn S.J. (2018). Current characterization methods for cellulose nanomaterials. Chem. Soc. Rev..

[51] Gharehkhani S., Sadeghinezhad E., Kazi S.N., Yarmand H., Badarudin A., Safaei M.R., Zubir M.N.M. (2015). Basic effects of pulp refining on fiber properties—A review. Carbohydr. Polym..

[52] Falcoz-Vigne L., Ogawa Y., Molina-Boisseau S., Nishiyama Y., Meyer V., Petit-Conil M., Mazeau K., Heux L. (2017). Quantification of a tightly adsorbed monolayer of xylan on cellulose surface. Cellulose.

[53] Toivonen M.S., Kurki-Suonio S., Schacher F.H., Hietala S., Rojas O.J., Ikkala O. (2015). Water-resistant, transparent hybrid nanopaper by physical cross-linking with chitosan. Biomacromolecules.

[54] Hoenig E., Winkler T., Mielke G., Paetzold H., Schuettler D., Goepfert C., Machens H.-G., Morlock M.M., Schilling A.F. (2011). High amplitude direct compressive strain enhances mechanical properties of scaffold-free tissue-engineered cartilage. Tissue Eng. Part A.

[55] Ayturk U.M., Garcia J.J., Puttlitz C.M. (2010). The micromechanical role of the annulus fibrosus components under physiological loading of the lumbar spine. J. Biomech. Eng..

[56] McMillan D.W., Garbutt G., Adams M.A. (1996). Effect of sustained loading on the water content of intervertebral discs: Implications for disc metabolism. Ann. Rheum. Dis..

[57] Nerurkar N.L., Mauck R.L., Elliott D.M. (2011). Modeling interlamellar interactions in angle-ply biologic laminates for annulus fibrosus tissue engineering. Biomech. Model. Mechanobiol..

[58] Lin N., Huang J., Dufresne A. (2012). Preparation, properties and applications of polysaccharide nanocrystals in advanced functional nanomaterials: A review. Nanoscale.

